# Effects of vitamin D supplementation on carotid intima-media thickness in HIV-infected youth

**DOI:** 10.1080/21505594.2017.1365217

**Published:** 2017-10-05

**Authors:** Allison Ross Eckard, Paolo Raggi, Mary Ann O'Riordan, Julia C. Rosebush, Danielle Labbato, Ann Chahroudi, Joshua H. Ruff, Christopher T. Longenecker, Vin Tangpricha, Grace A. McComsey

**Affiliations:** a Medical University of South Carolina, Charleston, SC, USA; b Emory University School of Medicine, Atlanta, GA, USA; c Mazankowski Alberta Heart Institute and University of Alberta, Edmonton, Alberta, Canada; d Case Western Reserve University and Rainbow Babies & Children's Hospital, Cleveland, OH, USA

**Keywords:** cardiovascular disease, carotid intima-media thickness, HIV, pediatrics and adolescents, randomized-controlled trial, vitamin D

Viral suppression with combination antiretroviral therapy (cART) dramatically improves survival in HIV-infected individuals. Yet, this population has more age-related co-morbidities, especially cardiovascular disease (CVD), and these co-morbidities occur at an earlier age than in the general population.^1^ Similar to their adult counterparts, data show that HIV-infected youth are also at an increased risk of development of these HIV-related co-morbidities later in life, despite few clinical manifestations in younger age.^2^ Developing complementary strategies to cART aimed at decreasing the risk of HIV-associated co-morbidities before clinical manifestations develop may greatly improve quality and life expectancy in this vulnerable population.

Vitamin D supplementation is arguably a potential adjuvant to cART based on the currently available data in both the HIV and general populations. A number of studies have shown that vitamin D deficiency independently increases the risk of CVD in the general population.^3^ In addition, many studies have shown an independent inverse association between serum 25-hydroxyvitamin D (25(OH)D) concentrations, an established marker of overall vitamin D status,^4^ and carotid intima-media thickness (IMT) in a variety of patient populations.^5-7^ Carotid IMT is considered a marker of subclinical atherosclerosis and has been associated with cardiovascular events and all-cause mortality in the general population.^8^ We and others have shown that low vitamin D status is independently associated with higher carotid IMT in HIV-infected adults.^9,10^

The proposed mechanisms whereby vitamin D contributes to CVD development or progression include its inhibitory effects on inflammatory cytokines,^11^ improved glycemic control and insulin sensitivity,^12^ direct effects on the vasculature,^13^ and inhibition of the renin-angiotensin-II aldosterone system.^14^ Vitamin D may play a particularly important role in HIV-related CVD, as many of these proposed mechanisms are similar to the pathophysiology that contributes to HIV-related CVD. In HIV, the increased CVD risk is multifactorial and thought to be related to increased inflammation/immune activation, endothelial dysfunction, and metabolic abnormalities such as insulin resistance.^15^

However, the few randomized-controlled trials (RCTs) evaluating the effect of vitamin D supplementation on lowering blood pressure and CVD risk in the general population have given conflicting and inconclusive results.^16-19^ In HIV, there have been few vitamin D supplementation RCTs and none have investigated changes in carotid IMT or CVD risk. Limited RCT data suggest that vitamin D supplementation may improve immune function and/or decrease immune activation in HIV, making the case to investigate its effect on CVD risk.^20-24^

In this current investigation, we present the effect of 24 months of vitamin D supplementation on carotid IMT in HIV-infected, cART-suppressed children and young adults in the context of a RCT. Our focus on HIV-infected youth represents an innovative approach to potentially identify efficacious strategies to prevent the development of HIV-related co-morbidities before the onset of established disease.

This is a randomized, active-control, double-blind, 24-month trial designed to measure the effect of vitamin D supplementation in HIV-1-infected youth. Participants were recruited from the HIV clinics of University Hospitals Case Medical Center, Cleveland, OH and Grady Health System, Atlanta, GA via electronic medical record system queries and case manager/provider referrals. Participants were eligible if they were between 8–25 y of age with documented HIV-1 infection on a stable cART regimen for ≥ 12 weeks, with ≥ 6 months cumulative cART duration, HIV-1 RNA level <1,000 copies/mL, with no intent to change cART regimen, diet, sun exposure or exercise routine during the study period, and a baseline serum 25(OH)D concentration ≤ 30 ng/mL (the Endocrine Society's current definition of vitamin D sufficiency is ≥ 30 ng/mL^25^). Exclusion criteria included routine vitamin D supplementation >400 IU/day, acute illness or inflammatory condition, malignancy, parathyroid or calcium disorder, diabetes, creatinine clearance <50 mL/min, liver enzymes ≥ 2.5 times the upper limit of normal, hemoglobin ≤ 9.0 g/dL, medication use (*e.g.*, chemotherapy agents, systemic steroids) which could affect results, or unwillingness/inability to comply with study procedures.

A healthy uninfected group was enrolled in a similar parallel study for comparison. The healthy uninfected group comprised subjects 8–25 y of age who self-reported no chronic diseases or current/recent illnesses. Healthy uninfected subjects were recruited aiming to achieve a group with similar characteristics (in terms of median age and proportion of males and black subjects) to the HIV-infected subjects. Healthy uninfected subjects were recruited in multiple ways, including: a) friends or family members of the HIV-infected subjects, b) physician referrals from local pediatric and adult clinics, c) extensive outreach to various local organizations, churches, and schools, and d) recruitment flyers in targeted locations throughout the 2 cities. Healthy uninfected subjects were recruited from similar geographic and socioeconomic areas as the HIV-infected group, to decrease the risk of potential confounders. HIV-negative status was confirmed with OraQuick Advance Rapid HIV test (OraSure Technologies, Inc., Bethlehem, PA) for subjects ages 12 and older at the Emory University site, given the high prevalence of HIV in this age group in Atlanta, Georgia.

Both HIV-infected subjects and healthy uninfected subjects were excluded from the study if they were pregnant or lactating.

Intervention consisted of 3 different monthly vitamin D_3_ (cholecalciferol) doses [18,000 IU/month (standard/control dose); 60,000 IU/month (moderate dose); 120,000 IU/monthly (high dose)] (Tischon Corp., Salisbury, MD). Doses were chosen to represent an approximate monthly equivalent to 600, 2,000, or 4,000 IU/daily, respectively. Six hundred IU/daily is the current Institute of Medicine's (IOM) recommended dietary allowance (RDA) of vitamin D across our study population. This amount is considered sufficient by the IOM Food and Nutrition Board to meet the requirements of 97.5% of healthy individuals in each life-stage and sex group.^26^ The monthly bolus dosing strategy was designed to minimize additional pill burden, given the risk of poor adherence to medication among adolescents and young adults. Serum 25(OH)D, the major circulating form of vitamin D and the metabolite most commonly used to assess overall vitamin D status, has a long half-life of ∼15 days,^27^ theoretically allowing a longer interval between vitamin D doses.

The randomization scheme was computer-generated, stratified by efavirenz (EFV) use at entry (this antiretroviral drug has been shown to affect 25(OH)D concentrations in some studies^28,29^) and study drugs were provided by the investigational pharmacy departments at each of the 2 sites. Regardless of randomization, participants took 2 capsules of vitamin D_3_ orally at baseline and then monthly after being prompted by a reminder phone call from study staff; capsules looked identical regardless of dose. Participants returned for study visits every 3 months throughout the duration of the study. These study visits were not timed in any way to the timing of when the subject took his/her monthly vitamin D dose. Representative capsules were sent to an independent laboratory (Analytical Research Laboratories, Oklahoma City, OK) at regular intervals during the study period to ensure continued potency of each dose.

The study was reviewed and approved by the Institutional Review Boards of University Hospital Case Medical Center, Emory University and Grady Health System. Parents or legal guardians gave informed consent for minors, and patients ≥ 18 y of age signed their own informed consent. The study was registered on clinicaltrials.gov (NCT01523496).

Here we present the pre-planned analysis that assessed changes in carotid IMT from baseline to 24 months in HIV-infected subjects and healthy uninfected subjects.

For all subjects, relevant data were obtained by questionnaire, including demographics, current and past medical history, alcohol intake, tobacco use, and drug habits. Further information was also collected from the HIV-infected subjects' medical records including past and current medical diagnoses, CD4 nadir, detailed past and current antiretroviral (ARV) and non-ARV medication use, HIV diagnosis date, and acquisition method (perinatal or horizontal). Targeted physical examination, weight and height, and blood pressure measurements were obtained in all subjects.

Blood was collected from all participants after at least an 8-hour fast. Serum concentrations of 25(OH)D were measured as the best measure of overall vitamin D status.^4^ Serum 25(OH)D concentrations were measured using either an automated chemiluminescent technique (IDS-iSYS automated machine, Immunodiagnostic Systems, Inc., Fountain Hills, AZ for Emory site) or a competitive immunoassay (ADVIA Centaur XP System, Siemens Healthcare Diagnostics, Inc., Tarrytown, NY for Case site). Parathyroid hormone (PTH) was measured via ELISA (Immutopics, Inc., Athens, OH).

Fasting lipoprotein profiles, insulin and glucose were also measured. Insulin resistance was calculated using the homeostasis model assessment of insulin resistance (HOMA-IR; [fasting glucose (mg/dl) × fasting insulin (mU/ml)]/405).^30^ Absolute CD4+ T-cell count and plasma HIV-1 RNA level were concomitantly measured as markers of HIV disease activity in the HIV-infected subjects.

For all laboratory assessments, laboratory personnel were blinded to clinical information and HIV status.

Carotid ultrasound was performed on all enrolled subjects at entry and at the 24-month study visit. Carotid intima-media thickness (IMT) was measured bilaterally at the level of the common carotid artery (CCA), internal carotid artery (ICA), and carotid bulb, using a Philips iU22 ultrasound system with a L9–3 MHz linear array transducer according to the consensus protocol of the American Society of Echocardiography.^31^ Ten-second cine-loops were obtained of the distal 1 cm of the CCA at 3 separate angles (anterior, lateral, and posterior) and of the ICA and carotid bulb at the optimum angle of insonation bilaterally. The far wall IMT was then measured offline using semi-automated edge detection software (Medical Imaging Applications LLC, Coralville, IA). The CCA measurements from the left side were averaged to produce a single mean measurement for that side. This process was repeated for the right side. Then, the measurements from the left and right side were averaged to produce a single mean. This process was then repeated for both the ICA and bulb IMT. All images were reviewed by a cardiologist (CTL) who was blinded to the subjects' HIV status.

Analyses were performed using intent-to-treat principles based on randomized treatment assignment that used all available data. Variables are described first by each study group (all HIV-infected subjects vs. all healthy uninfected subjects).

Data from the moderate- and high-dose arms within the HIV-infected subjects were first analyzed. It was determined in preliminary analyses that there were no statistically significant IMT changes within either of these arms and no significant differences in IMT changes between arms over the study period. Thus, the subjects randomized to receive the moderate or high dose were considered together (supplementation arm) and compared with subjects randomized to receive the standard dose (standard arm).

The process was then repeated within the healthy uninfected group, where subjects randomized to receive the moderate or high dose were considered together (supplementation arm) and compared with subjects randomized to receive the standard dose (standard arm).

Continuous measures are described by medians and interquartile ranges, and nominal variables are described with frequencies and percentages. Nominal variables were compared using χ^2^ analysis or Fisher's exact test. Continuous measures were tested for normality. For between-group comparisons (baseline and changes from baseline to 24 months), normally-distributed variables were compared using the *t*-test, and non-normally-distributed variables were compared using Wilcoxon rank sum test. For within-group changes from baseline to 24 months, normally-distributed variables were compared with the paired *t*-test, and non-normally-distributed variables were compared with Wilcoxon signed rank test.

Correlations between changes in 25(OH)D concentrations and variables of interest were assessed using Spearman correlation coefficients for continuous variables. Appropriate 2-sample tests were used to assess marker differences in sub-groups for dichotomous variables (*e.g.*, protease inhibitor (PI) use at entry yes vs. no).

Multivariable regression analyses were used to determine variables independently associated with changes in carotid IMT within the HIV-infected subjects. Variables were selected for inclusion in the regression models based on the results of the bivariate analyses or clinical relevance. The effect of vitamin D supplementation on carotid IMT was considered by analyzing the following 2 variables in separate regression models: 1) treatment group (supplementation-dose arm vs. standard-dose arm) and 2) change in serum 25(OH)D concentration over 24 months regardless of treatment group.

All statistical tests were 2-sided with a 0.05 significance level. Analyses were performed with SAS version 9.4 (SAS Institute, Cary, NC).

One hundred and 2 HIV-infected subjects and 88 healthy uninfected subjects were enrolled between January 2012 and September 2014 (see Fig. S1). Sixty-eight HIV-infected subjects and 54 healthy uninfected subjects completed their 24-month visit and were included in the current analysis.

Among the HIV-infected subjects, there were no statistically significant differences between the supplementation-dose and standard-dose arms for any of the baseline variables except that the body mass index (BMI)was higher in the supplementation-dose arm and there were more current smokers (any amount of smoking) vs. the standard-dose arm. There was no difference between arms as far as CCA and ICA IMT, but the standard-dose arm had a statistically higher carotid bulb IMT compared with the supplementation-dose arm (median (Q1, Q3) = 0.66 (0.60, 0.72) mm vs. 0.59 (0.54, 0.66) mm, respectively; P = 0.04) (see Table S1 for baseline characteristics).

As designed, all HIV-infected subjects had a low number of HIV-1 RNA copies (< 1,000 copies/mL). Because there were several different assays used in various clinical laboratories to measure HIV-1 RNA levels, there were varying lower limits of detection (< 20, <40, <48, <79 and <80 copies/mL), and 90% of the subjects had < 80 HIV-1 RNA copies/mL. Of the 7 remaining subjects, the median HIV-1 RNA level was 145 copies/mL.

At baseline, 26 subjects were on a ritonavir-boosted PI (darunavir = 20; atazanavir = 13; lopinavir = 5), 25 subjects were on a non-nucleoside reverse transcriptase inhibitor (NNRTI) (EFV = 16; rilpivirine = 6; nevirapine = 2; etravirine = 1), and 5 subjects were on an integrase inhibitor (elvitegravir/cobicistat = 4; raltegravir = 1). Nucleoside reverse transcriptase inhibitor (NRTI) backbones included emtricitabine/tenofovir (N = 59), lamivudine/abacavir (N = 5), lamivudine/zidovudine (N = 2), stavudine/abacavir (N = 1), and lamivudine/zidovudine/abacavir (N = 1).

The randomized groups (supplementation-dose and standard-dose arms) within the healthy uninfected subjects were well-balanced except that the low-density lipoprotein (LDL) cholesterol and PTH were significantly higher in the supplementation-dose arm compared with the standard-dose arm.

The HIV-infected and healthy uninfected groups were well-matched for age, sex, race, and BMI, but there were more subjects in the HIV-infected group with Tanner stage 5. The HIV-infected group also had a significantly lower high-density lipoprotein (HDL) cholesterol and higher triglycerides and PTH levels compared with the healthy uninfected group. Bulb IMT measurements were significantly higher in the HIV-infected group compared with the healthy uninfected group with a trend toward significance with CCA IMT, but there were no statistically significant differences in ICA IMT. Additionally, insulin resistance as measured by HOMA-IR was similar between groups. Of note, none of the healthy uninfected subjects had a history of *in-utero* HIV/ARV exposure. Only 4 of the healthy uninfected subjects had a biologic relationship with the HIV-infected subjects.

For the HIV-infected and uninfected healthy subjects combined, there were no differences in median (Q1, Q3) serum 25(OH)D concentrations between subjects at the 2 different sites at baseline (Case: 16 (13, 20) ng/mL, Emory: 18 (14, 24) ng/mL; P = 0.08). The percentage of subjects enrolled during summer months (June–August) was similar at the 2 sites (Emory: 20%; Case 27%). There were also no differences based on sex at baseline (male: 17 (14, 21) ng/mL; female: 18 (13, 23) ng/mL; P = 0.97).

During the 24-month study period, 16 HIV-infected subjects changed ART regimens, reflecting in part updates in the *Guidelines for the Use of Antiretroviral Agents in HIV-1-infected Adults and Adolescents*.^32^ Of note, 6 subjects stopped EFV (3 from each study arm) and changed to dolutegravir (N = 1), elvitegravir/cobicistat (N = 2), rilpivirine (N = 2), or darunavir/ritonavir (N = 2). One subject stopped the PI-based regimen and started EFV, and one subject stopped the PI-based regimen, took EFV for several months and then re-started the same PI (both from standard-dose arm). There were no changes in any of these subjects NRTI backbone regimen. One additional subject stopped all ARVs ∼6 months before the 24-month visit. Eighty-one percent of HIV-infected subjects still enrolled at 24 months maintained an HIV-1 RNA level <1000 copies/mL throughout the study period.

Within the HIV-infected subjects, 25(OH)D concentrations increased significantly in both the supplementation-dose and standard-dose arms but with a greater increase in the supplementation-dose arm (Table 1A).

**Table 1.** Characteristics During Study Period.

A. Changes over 24 Months in HIV-infected Subjects								
Median (Q1, Q3)	All Subjects (N = 68)	P^°^	Supplementation Dose^*^ (N = 42)	P^°^	Standard Dose^**^ (N = 26)	P^°^	P^†^	
*Clinical and Laboratory Variables*								
BMI, kg/m^2^	+1.1 (+0.1, +2.3)	<0.0001	+1.0 (-0.2, +2.9)	0.0001	+1.4 (+0.6, +1.9)	<0.0001	0.82	
Waist-to-hip ratio	+0.01 (-0.04, +0.04)	0.80	+0.01 (-0.03, +0.05)	0.49	−0.00 (-0.06, +0.03)	0.53	0.43	
CD4 count, cells/mm^3^	+42 (-85, +159)	0.07	+57 (-77, +186)	0.07	+24 (-85, +158)	0.29	0.49	
Systolic BP, mmHg	+4 (-5, +9)	0.12	+4 (-5, +10)	0.16	+3 (-5, +5)	0.39	0.60	
LDL cholesterol, mg/dL	−6 (-19, +3)	0.003	−8 (-19, -1)	0.01	−5 (-18, +3)	0.12	0.57	
HDL cholesterol, mg/dL	+2 (-4, +6)	0.35	0 (-4, +5)	1.00	+4 (-1, +7)	0.16	0.17	
Triglycerides, mg/dL	+2.5 (-20, +19)	0.95	+8 (-26, +21)	0.79	−1 (-16, +17)	0.74	0.63	
HOMA-IR	−0.01 (-0.89, +0.96)	0.64	+0.13 (-0.75, +1.56)	0.43	−0.06 (-1.27, +0.73)	0.80	0.42	
25(OH)D, ng/mL	+18 (+9, +25)	<0.0001	+23 (+13, +33)	<0.0001	+3 (-4.5, +5.0)	<0.0001	0.004	
*Carotid IMT*								
CCA IMT, mm	−0.01 (-0.06, +0.02) (N = 67)	0.06	−0.01 (-0.05, +0.02) (N = 42)	0.44	−0.02 (-0.08, +0.02) (N = 25)	0.06	0.26	
Bulb IMT, mm	−0.06 (-0.21, +0.02) (N = 53)	0.0006	−0.03 (-0.13, +0.04) (N = 35)	0.11	−0.19 (-0.27, -0.03) (N = 18)	0.0008	0.01	
ICA IMT, mm	−0.04 (-0.17, +0.04) (N = 40)	0.10	−0.03 (-0.06, +0.03) (N = 26)	0.30	−0.09 (-0.19, +0.04) (N = 14)	0.15	0.34	
B. Changes over 24 Months in Healthy Uninfected Subjects								
Median (Q1, Q3)	All Subjects (N = 54)	P^°^	Supplementation Dose^*^(N = 33)	P^°^	Standard Dose^**^ (N = 21)	P^°^	P^†^	P^‡^
*Clinical and Laboratory Variables*								
BMI, kg/m^2^	+1.4 (+0.0, +2.78)	<0.0001	+1.4 (+0.0, +2.8)	0.001	+1.5 (+0.0, +2.8)	0.01	0.79	0.95
Waist-to-hip ratio	+0.00 (-0.01, +0.03)	0.44	+0.00 (-0.01, +0.03)	0.70	+0.00 (-0.02, +0.02)	0.48	0.83	0.96
Systolic BP, mmHg	+6 (+2, +15)	<0.0001	+7 (+2, +14)	<0.0001	+6 (+1, +15)	0.005	0.70	0.01
LDL cholesterol, mg/dL	−2 (-19, +6)	0.15	−7 (-20, +1)	0.06	+0 (-12, +14)	0.90	0.13	0.43
HDL cholesterol, mg/dL	−2 (-5, +6)	0.44	−2 (-4, +6)	0.96	+0 (-7, +3)	0.35	0.51	0.23
Triglycerides, mg/dL	+0.(-12, +22)	0.38	+8 (-7, +27)	0.09	−4 (-15, +9)	0.46	0.06	0.61
HOMA-IR	+0.12 (-0.88, +1.27)	0.71	+0.33 (-0.97, +1.36)	0.73	+0.11 (-0.50, +0.52)	0.78	0.78	0.96
25(OH)D, ng/mL	+14 (+4, +23)	<0.0001	+21 (+12, +32)	<0.0001	+4 (+0, +12)	0.0003	<0.0001	0.17
*Carotid IMT*								
CCA IMT, mm	−0.01 (-0.07, +0.02) (N = 52)	0.07	−0.01 (-0.07, +0.02) (N = 32)	0.22	−0.02 (-0.06, +0.02) (N = 20)	0.14	0.69	0.82
Bulb IMT, mm	−0.03 (-0.13, +0.04) (N = 44)	0.06	−0.03 (-0.12, +0.06) (N = 28)	0.33	−0.03 (-0.16, +0.04) (N = 16)	0.08	0.71	0.23
ICA IMT, mm	−0.03 (-0.15, +0.07) (N = 33)	0.07	−0.05 (-0.15, +0.07) (N = 20)	0.22	−0.02 (-0.22, +0.07) (N = 13)	0.27	0.88	0.97
C. Variables at 24 Months in HIV-infected Subjects								
Median (Q1, Q3)	All Subjects (N = 68)		Supplementation Dose^*^ (N = 42)		Standard Dose^**^ (N = 26)		P^†^	
25(OH)D, ng/mL	37 (28, 44)		41 (31, 46)		32 (25, 38)		0.006	
CCA IMT, mm	0.55 (0.51, 0.58)		0.56 (0.51, 0.58)		0.54 (0.49, 0.57)		0.39	
Bulb IMT, mm	0.54 (0.47, 0.58)		0.55 (0.50, 0.60)		0.48 0.43, 0.56)		0.02	
ICA IMT, mm	0.50 (0.39, 0.57)		0.51 (0.39, 0.57)		0.50 (0.40, 0.57)		0.98	
D. Variables at 24 Months in Healthy Uninfected Subjects								
Median (Q1, Q3)	All Subjects (N = 54)		Supplementation Dose^*^ (N = 33)		Standard Dose^**^ (N = 21)		P^†^	^‡^P ^‡^
25(OH)D, ng/mL	30 (23, 39)		38 (29, 51)		24 (20, 30)		0.0005	0.046
CCA IMT, mm	0.54 (0.51, 0.57)		0.55 (0.48, 0.58)		0.53 (0.51, 0.55)		0.36	0.17
Bulb IMT, mm	0.54 (0.42, 0.58)		0.39 (0.20, 0.54)		0.53 (0.43, 0.56)		0.49	0.52
ICA IMT, mm	0.49 (0.32, 0.54)		0.44 (0.32, 0.54)		0.50 (0.28, 0.54)		0.91	0.14

*Notes*.

* Supplementation dose = 60,000 IU/month (moderate dose) or 120,000 IU/month (high dose);

** Standard dose = 18,000 IU/month (control dose)

° P value for changes within group;

† P value for differences in changes between the 2 dosing arms;

‡ P value for differences in changes between combined HIV+ and combined healthy uninfected subjects; Q, quartile; BMI, body mass index; BP, blood pressure; LDL, low-density lipoprotein; HDL, high-density lipoprotein; HOMA-IR, homeostatic model assessment of insulin resistance; 25(OH)D, 25-hydroxyvitamin D; IMT, intima-media thickness; CCA, common carotid artery; ICA, internal carotid artery

Within the supplementation-dose arm, there were no statistically significant changes in carotid IMT. However, within the standard-dose arm, carotid bulb IMT decreased significantly (Fig. 1), and CCA IMT showed a trend toward a significant decrease. The comparison between the standard-dose arm and the supplementation-dose arm as far as carotid bulb IMT change was statistically significant.

**Figure 1.**
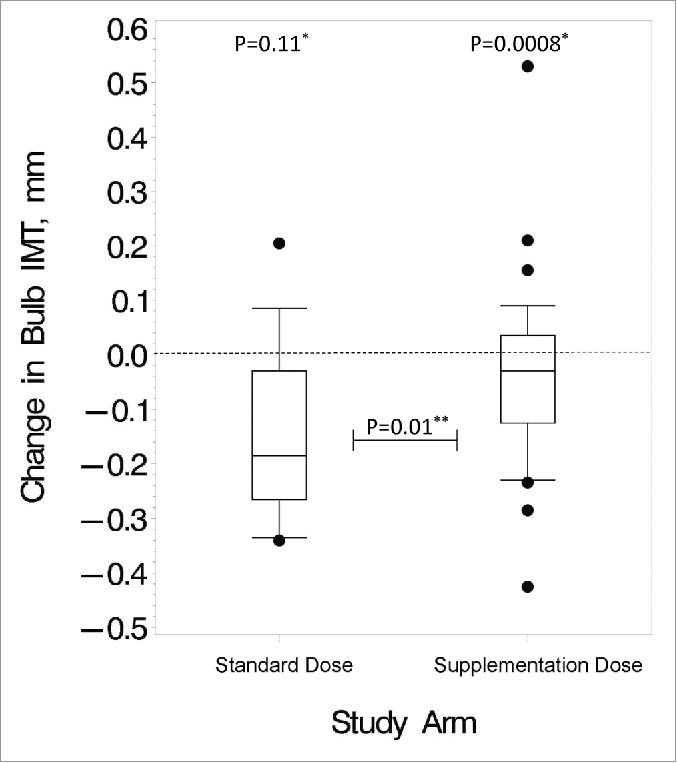
Changes in carotid bulb IMT over the study period. These box and whisker plots show the distribution of changes after 24 months in the standard vs. supplementation arm for the HIV-infected subjects. Bottom and top edges of the box represent 25^th^ and 75^th^ percentiles, respectively, the center horizontal line is drawn at the median, and the bottom and top vertical lines (whiskers) extend to the 10^th^ and 90^th^ percentiles, respectively. Small circles outside of the vertical lines represent outlier data points. *P value for changes within group; **P value for differences in changes between the 2 dosing arms

In terms of CVD risk factors, there was a significant decrease in LDL cholesterol within the supplementation arm not seen in the standard arm. However, there were no significant differences in the changes between the 2 arms. There were no significant changes in waist-to-hip ratio, HDL cholesterol, triglycerides, or HOMA-IR for either dosing arm. Increases in BMI were seen in both groups. The increase in CD4 count trended toward significance within the supplementation arm.

Among the healthy uninfected group, increases in 25(OH)D concentrations followed patterns similar to those of the HIV-infected subjects (Table 1B). However, there were no statistically significant changes in carotid IMT within the healthy uninfected group for either the supplementation-dose or standard-dose arms, and systolic blood pressure increased significantly in both arms.

There were few significant differences in the changes between the HIV-infected subjects and healthy uninfected group, except the increases in systolic blood pressure were significantly more in the uninfected group (Table 1B). There were no differences in the changes for BMI, waist-to-hip ratio, LDL cholesterol, HDL cholesterol, triglycerides, HOMA-IR, 25(OH)D, and carotid IMT. There was a trend toward a higher 25(OH)D serum concentration at 24 months in the HIV-infected group compared with the uninfected group (Table 1D).

For the HIV-infected and uninfected healthy subjects combined, there were no differences in median (Q1, Q3) serum 25(OH)D concentrations between subjects at the 2 different sites at 24 months (Case: 32 (24, 42) ng/mL, Emory: 36 (28, 45) ng/mL; P = 0.11). There were also no differences based on sex at 24 months (male: 35 (25, 44) ng/mL; female: 31 (25, 43) ng/mL; P = 0.76).

Within the HIV-infected group, changes in serum 25(OH)D concentrations were positively correlated with baseline CD4 count (R = 0.34; P = 0.01), ARV duration (R = 0.29; P = 0.045, PI duration (R = 0.32; P = 0.045), and changes in carotid bulb IMT (R = 0.43; P = 0.001) (Fig. 2A). The correlation between changes in carotid bulb IMT and changes in 25(OH)D concentrations was analyzed separately by dosing arm in the HIV-infected group (Fig. 2B). The correlation between the 2 variables was not significant within the standard-dose arm (R = -0.05; P = 0.86); however, the correlation remained statistically significant within the supplementation-dose arm (R = 0.47; P = 0.004).

**Figure 2.**
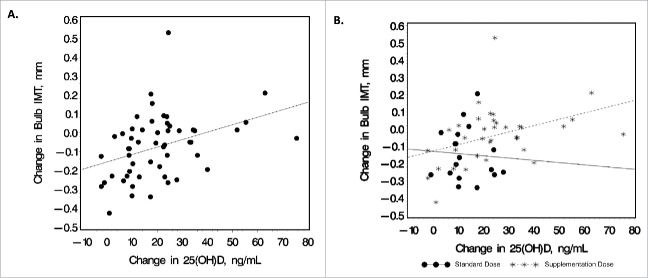
Relationship between change in 25(OH) and carotid bulb IMT. Scatter plots depict the bivariate relationship between changes in serum 25(OH)D concentrations and changes in carotid bulb IMT for (A). all HIV-subjects combined (R = 0.43; P = 0.001) and (B) shown by dosing arm separately (standard-dose arm: R = -0.05, P = 0.86; supplementation-dose arm: R = 0.47, P = 0.004). Increases in carotid bulb IMT were significantly correlated with increases in serum 25(OH)D concentrations, with this association driven by subjects in the supplementation arm. R, Spearman correlation coefficient. IMT, intima-media thickness; 25(OH)D, 25-hydroxyvitamin D

None of the other variables tested within the HIV-infected subjects showed a significant association with change in serum 25(OH)D concentrations. The variables considered included demographics, baseline or changes in CVD risk factors (sex, smoking, blood pressure, BMI, waist-to-hip ratio, lipid levels, HOMA-IR), changes in CCA and ICA IMT, HIV/ART factors (cumulative use of EFV, nadir CD4, HIV duration, PI use at entry, previous AIDS diagnosis, current use of EFV), alcohol use, and Tanner stage 5 vs. <5.

Variables of interest were included in 2 multivariable regression models to investigate changes in carotid bulb IMT in the HIV-infected group (see Table S2). The first model considered the effect of vitamin D supplementation on carotid IMT changes by investigating the study arm (supplementation dose vs. standard dose). The subjects randomized to the standard dose showed statistically significant decreases in carotid bulb IMT (P = 0.03). In addition, any amount of current smoking was also associated with greater decreases in carotid bulb IMT. In the second model, changes in 25(OH)D concentrations were considered as a continuous variable. Here, increases in 25(OH)D concentrations showed a trend toward significance where a greater increase in 25(OH)D concentration was associated with a greater increase in carotid bulb IMT (P = 0.08).

To our knowledge, this is the first RCT of vitamin D supplementation to investigate changes in carotid IMT and CVD risk in HIV-infected youth. Among the HIV-infected subjects, median serum 25(OH)D concentrations increased significantly after 24 months of monthly oral vitamin D_3_ for both study arms. However, serum 25(OH)D concentrations increased significantly more in the moderate- and high-dose supplementation arm. Surprisingly, carotid IMT did not change significantly within supplementation-dose arm, but decreased significantly in the bulb region and approached significance in the CCA within the standard-dose arm. In bivariate analyses, increases in carotid bulb IMT were significantly correlated with increases in serum 25(OH)D concentrations, with this association driven by subjects in the supplementation-dose arm. Moreover, being in the supplementation-dose arm was independently associated with increases in carotid bulb IMT.

As expected and consistent with our prior data,^33^ HIV-infected youth had increased CCA, ICA, and carotid bulb IMT at baseline when compared with the healthy uninfected subjects. Healthy uninfected subjects had similar increases in serum 25(OH)D concentrations as the HIV-infected subjects over the study period, except that there was a trend toward a higher serum 25(OH)D concentration in the HIV-infected subjects at 24 months. In contrast with HIV-infected youth, the healthy uninfected subjects did not demonstrate any significant changes in carotid IMT. The relationship between changes in carotid IMT and serum 25(OH)D concentrations observed in the HIV-infected group were not seen among the healthy uninfected subjects.

Our findings suggest that a standard dose of vitamin D_3_ given monthly over 24 months to HIV-infected subjects with vitamin D insufficiency decreases carotid IMT and potentially CVD risk, compared with a relatively high dose. In contrast, moderate and high doses appear to be independently associated with higher carotid IMT, and, likewise, potentially a higher CVD risk. This surprising finding is in contrast with several studies in the general population which showed an inverse relationship between serum 25(OH)D concentrations and carotid IMT.^5-7^ However, many of the published studies are cross-sectional in nature, and there are perhaps an equal number of studies that have failed to show such an association.^34-38^ In a meta-analysis which included only prospective studies, baseline circulating 25(OH)D was inversely associated with the risk of CVD in the majority, but not all studies.^3^ However, it is difficult to directly compare these studies to our current RCT.

Perhaps more relevant to our current findings are several studies that demonstrated a non-linear association between 25(OH)D concentrations and CVD risk.^39,40^ For example, van Dijk, *et al*^49^ found that there was a slight increase of carotid IMT with increasing 25(OH)D in an elderly population when the serum 25(OH)D concentration increased over 22.7 ng/mL (56.7 nmol/L). This finding could explain our unexpected results. However, the authors proposed that the potentially deleterious effect of high 25(OH)D concentrations could be secondary to increased vascular calcification as a result of higher circulating calcium levels caused by increased calcium absorption in the gastrointestinal tract. In our study, carotid IMT measurements were only taken where the vessel wall was free of plaque, and we did not see any significant changes in any group in serum calcium or parathyroid hormone levels (data not shown). However, vitamin D has the potential to influence the activity of about 3000 genes through upregulation of the vitamin D receptor (VDR) and modulates numerous biologic functions, many of which have not yet been fully elucidated.^41,42^ Thus, there could be other pathways by which a high 25(OH)D concentration could negatively impact CVD risk.

On the other hand, Gibson, *et al*^43^ provides evidence that high-dose vitamin D may actually be beneficial for reducing CVD risk. The authors showed that the assumed inactive sterol, vitamin D_3_ (as well as its metabolites 25(OH)D_3_ and 1α,25-dihydroxyvitamin D_3_ (1,25(OH)_2_D_3_)), is a potent and general mediator of endothelial stability through a non-genomic pathway. Vitamin D acts directly on the endothelium to inhibit permeability induced by diverse pro-inflammatory cues, thereby stabilizing its barrier structure and function and reducing vascular leak into surrounding tissues. This alternative signaling modality by vitamin D_3_, coupled with the known immunomodulatory effects of 1,25(OH)_2_D_3_,^44^ may explain why carotid IMT decreased in the standard-dose arm but did not change in the supplementation-dose arm within the HIV-infected group. It may also explain the absence of carotid IMT change among the healthy uninfected subjects, who do not have the same level of inflammation, immune activation and immune dysfunction known to affect patients with chronic HIV.^45^

Our study had some limitations worth mentioning, especially the heterogeneous nature of the HIV-infected subjects and the small sample size on which we measured carotid IMT within each dosing arm. This may explain why we observed statistically significant changes within the carotid bulb region, an area known to change more rapidly in HIV.^46^ Perhaps the most important limitation is the recognized difficulty in assessing carotid IMT longitudinally in youth. As Fernhall, *et al*^47^ pointed out in a thorough review of the literature, discrepancies among various studies of IMT in children may be due to the fact that IMT changes very little during childhood, and, as it changes, so does arterial size and luminal diameter.^48,49^ Thus, the carotid IMT changes observed over this 24-month study period may be clinically insignificant. A further limitation is the selection of most black HIV-infected subjects and the potential for a race-to-drug interaction that we could not address due to the small sample size.

It is also important to note that our study design used an active-control arm instead of a true placebo which likely blunted our ability to detect differences among the dosing arms. However, due to ethical concerns about failing to treat subjects with vitamin D insufficiency/deficiency, we chose not to have a true control/placebo arm. Likewise, we used a bolus dosing strategy designed to minimize additional pill burden, given the risk of poor adherence to medication among adolescents and young adults. Some data suggest that a daily dosing schedule may exert superior therapeutic effects compared with large bolus doses, which can result in both a steep and rapid increase in circulating 25(OH)D concentrations, followed by a slow decline.^50^ Thus, while bolus dosing strategies are particularly appealing in this population who often struggle with adherence to daily medications, daily dosing may be necessary to achieve clinically-meaningful benefits from vitamin D supplementation. Nevertheless, we believe our results are novel and deserve further investigation.

In conclusion, our study showed that a standard vitamin D_3_ dose of 18,000 IU/month given over 24 months resulted in a significant decrease in carotid bulb IMT compared with high monthly doses of 60,000 or 120,000 IU in HIV-infected youth. These results may suggest a potential positive effect of standard-dose vitamin D supplementation in this population, as a similar effect that was not seen among healthy uninfected youth; however, confirmatory studies are needed before making such conclusions. Further analyzes elucidating the direct and indirect mechanisms by which vitamin D supplementation may affect CVD risk in HIV are also warranted.

## Supplementary Material

KVIR_S_1365217.zip
