# Supplementary material for: Effects of vitamin D supplementation on carotid intima-media thickness in HIV-infected youth
Source: Virulence. 2017 Oct 5;9(1):294–305. doi: 10.1080/21505594.2017.1365217 (PMC5955463; doi:10.1080/21505594.2017.1365217)
Supplement: KVIR_S_1365217.zip [file kvir-09-01-1365217-s001.zip › Table S1.docx]

**Table S1**

| **Baseline Study Characteristics** | | | | | | | | | | | | | |  |
| --- | --- | --- | --- | --- | --- | --- | --- | --- | --- | --- | --- | --- | --- | --- |
| **Median (Q1, Q3) or no. (%)** | **HIV-infected Subjects** | | | | **Healthy Uninfected Subjects** | | | | | | | **P^‡^** | |  |
|  | **All Subjects**  **(N=68)** | **Supplementation Dose^*^**  **(N=42)** | **Standard Dose^**^**  **(N=26)** | **P^†^** | **All Subjects**  **(N=54)** | | **Supplementation Dose^*^**  **(N=33)** | | **Standard Dose^**^**  **(N=21)** | | **P^†^** |  |  |  |
|  | | | | | | | | | | | | | |  |
| **Demographics and Clinical Variables** | | | | | | | | | | | | | |  |
| Age, years | 19.8 (15.3, 22.6) | 19.3 (15.0, 23.8) | 20.1 (15.6, 21.6) | 0.91 | 16.9 (12.4, 22.6) | | 17.4 (12.4, 23.1) | | 16.5 (12.9, 18.5) | | 0.55 | 0.10 | |  |
| Male sex | 45 (66%) | 29 (69%) | 16 (62%) | 0.53 | 28 (52%) | | 17 (52%) | | 11 (52%) | | 0.95 | 0.11 | |  |
| Black race | 60 (88%) | 24 (92%) | 36 (86%) | 0.70 | 47 (87%) | | 27 (82%) | | 20 (95%) | | 0.23 | 0.84 | |  |
| BMI, kg/m^2^ | 22.1 (19.1, 25.2) | 23.7 (20.1, 26.5) | 20.9 (17.4, 22.6) | **0.01** | 22.8 (19.3, 28.1) | | 23.7 (19.3, 28.1) | | 22.4 (19.4, 27.5) | | 0.57 | 0.47 | |  |
| Tanner stage 5 | 48 (71%) | 30 (71%) | 18 (69%) | 0.85 | 27 (50%) | | 18 (55%) | | 9 (43%) | | 0.40 | **0.02** | |  |
| Systolic BP, mmHg | 120 (112, 128) | 120 (114, 128) | 119 (104, 128) | 0.28 | 117 (106, 124) | | 118 (106, 124) | | 110 (1107, 122) | | 0.59 | 0.15 | |  |
| Current smoking^°^ | 11 (16%) | 10 (24%) | 1 (4%) | **0.04** | 7 (13%) | | 6 (18%) | | 1 (5%) | | 0.23 | 0.62 | |  |
| Current alcohol use^°^ | 29 (43%) | 16 (62%) | 10 (39%) | 0.53 | 14 (26%) | | 9 (26%) | | 5 (24%) | | 0.78 | 0.05 | |  |
| Waist-to-hip ratio | 0.85 (0.80, 0.89) | 0.85 (0.79, 0.91) | 0.84 (0.80, 0.86) | 0.32 | 0.82 (0.79, 0.88) | | 0.82 (0.80, 0.88) | | 0.83 (0.78, 0.89) | | 0.74 | 0.18 | |  |
| LDL cholesterol, mg/dL | 90 (72, 113) | 88 (72, 112) | 97 (71, 115) | 0.77 | 87 (70, 107) | | 95 (79, 109) | | 76 (61, 93) | | **0.02** | 0.44 | |  |
| HDL cholesterol, mg/dL | 47 (39, 56) | 46 (38, 54) | 48 (41, 56) | 0.39 | 55 (46, 65) | | 54 (47, 68) | | 56 (46, 63) | | 0.75 | **0.0001** | |  |
| Triglycerides, mg/dL | 86 (65, 118) | 88 (67, 135) | 75 (61, 96) | 0.22 | 59 (45, 83) | | 63 (48, 79) | | 48 (37, 93) | | 0.50 | **0.0002** | |  |
| HOMA-IR | 2.1 (1.5, 4.3) | 2.6 (1.5, 4.4) | 2.0 (1.4, 3.6) | 0.42 | 2.5 (1.5, 3.9) | | 2.4 (1.5, 3.9) | | 2.5 (1.7, 4.1) | | 0.88 | 0.94 | |  |
| 25(OH)D, ng/mL | 17 (14, 22) | 17 (14, 20) | 18 (13, 26) | 0.61 | 17 (13, 21) | | 17 (13, 20) | | 18 (13, 24) | | 0.40 | 0.78 | |  |
| PTH, pg/mL | 47.9 (16.4, 56.1) | 55.8 (47.0, 75.2) | 56.2 (51.0, 72.9) | 0.72 | 43.9 (34.4, 66.8) | | 51.7 (39.1, 62.4) | | 35.3 (26.5, 66.8) | | **0.03** | **0.03** | |  |
| **Carotid IMT** | | | | | | | | | | | | | |  |
| CCA IMT, mm | 0.56 (0.54, 0.59)  (N=68) | 0.56 (0.53, 0.59)  (N=42) | 0.57 (0.55, 0.59)  (N=26) | 0.68 | 0.55 (0.52, 0.57)  (N=53) | | 0.55 (0.51, 0.57)  (N=33) | | 0.56 (0.53, 0.58)  (N=20) | | 0.62 | 0.06 | |  |
| Bulb IMT, mm | 0.62 (0.54, 0.70)  (N=55) | 0.59 (0.54, 0.66)  (N=35) | 0.66 (0.60, 0.72)  (N=20) | **0.04** | 0.55 (0.50, 0.61)  (N=45) | | 0.55 (0.50, 0.62)  (N=29) | | 0.55 (0.53, 0.60)  (N=16) | | 0.94 | **0.002** | |  |
| ICA IMT, mm | 0.55 (0.47, 0.59)  (N=41) | 0.54 (0.40, 0.61)  (N=26) | 0.56 (0.50, 0.58)  (N=15) | 0.42 | 0.50 (0.38, 0.56)  (N=36) | | 0.49 (0.39, 0.56)  (N=22) | | 0.53 (0.38, 0.57)  (N=14) | | 0.75 | 0.17 | |  |
| **HIV Variables** | | | | | | | | | | | | | | |
| Current CD4, cells/mm^3^ | 654 (430, 894) | 656 (419, 899) | 654 (464, 872) | 0.85 | -- | -- | | -- | | -- | | | -- | |
| Nadir CD4, cells/mm^3^ | 290 (119, 424) | 318 (157, 474) | 255 (33, 389) | 0.34 | -- | -- | | -- | | -- | | | -- | |
| HIV RNA <80 copies/mL | 61 (90%) | 37 (88%) | 24 (92%) | 0.70 | -- | -- | | -- | | -- | | | -- | |
| HIV RNA, copies/mL | 145 (127, 590)  (N=7) | 143 (127, 190)  (N=5) | 533 (145, 920)  (N=2) | 0.33 | -- | -- | | -- | | -- | | | -- | |
| Perinatal transmission | 37 (54%) | 24 (57%) | 13 (50%) | 0.57 | -- | -- | | -- | | -- | | | -- | |
| Previous AIDS diagnosis | 27 (41%) | 17 (43%) | 10 (39%) | 0.74 | -- | -- | | -- | | -- | | | -- | |
| HIV duration, years | 9.6 (2.9, 15.7) | 10.5 (3.2, 16.5) | 7.2 (2.7, 14.5) | 0.34 | -- | -- | | -- | | -- | | | -- | |
| ARV duration, years | 6.0 (1.6, 10.1) | 6.6 (2.0, 10.5) | 3.2 (1.25, 9.5) | 9,39 | -- | -- | | -- | | -- | | | -- | |
| NRTI duration, years | 5.3 (1.6, 9.7) | 6.5 (2.0, 10.1) | 2.5 (1.3, 7.0) | 0.19 | -- | -- | | -- | | -- | | | -- | |
| PI duration, years | 3.0 (0.9, 8.2) | 4.1 (1.0, 8.5) | 2.0 (0.8, 6.5) | 0.41 | -- | -- | | -- | | -- | | | -- | |
| EFV duration, months | 0.5 (0.0, 1.4) | 0.6 (0.0, 1.6) | 0.0 (0.0, 1.0) | 0.09 | -- | -- | | -- | | -- | | | -- | |
| TDF duration, months | 1.6 (0.6, 2.7) | 1.6 (0.6, 2.8) | 1.6 (0.7, 9.5) | 0.92 | -- | -- | | -- | | -- | | | -- | |
| Current PI use | 29 (43%) | 16 (38%) | 13 (50%) | 0.34 | -- | -- | | -- | | -- | | | -- | |
| Current EFV use | 17 (25%) | 11 (26%) | 6 (23%) | 0.77 | -- | -- | | -- | | -- | | | -- | |
| Current TDF use | 51 (75%) | 33 (79%) | 18 (69%) | 0.39 | -- | -- | | -- | | -- | | | -- | |
| **^*^**Supplementation dose = 60,000 IU/month (moderate dose) or 120,000 IU/month (high dose); **^**^**Standard dose = 18,000 IU/month (control dose)  ^†^P value between supplementation and standard dosing arms; ^‡^P value between combined HIV+ and combined healthy uninfected subjects; Bold-faced P values designate those <0.05.  ^°^Any amount of consumption in the preceding 3 months  Q, quartile; BMI, body mass index; BP, blood pressure; LDL, low-density lipoprotein; HDL, high-density lipoprotein; HOMA-IR, homeostatic model assessment of insulin resistance; 25(OH)D, 25-hydroxyvitamin D; PTH, parathyroid hormone; IMT, intima-media thickness; CCA, common carotid artery; ICA, internal carotid artery; ARV, antiretroviral; NRTI, nucleoside reverse transcriptase inhibitor; PI, protease inhibitor; EFV, efavirenz; TDF, tenofovir | | | | | | | | | | | | | | |
