# Supplementary material for: Effects of vitamin D supplementation on carotid intima-media thickness in HIV-infected youth
Source: Virulence. 2017 Oct 5;9(1):294–305. doi: 10.1080/21505594.2017.1365217 (PMC5955463; doi:10.1080/21505594.2017.1365217)
Supplement: KVIR_S_1365217.zip [file kvir-09-01-1365217-s001.zip › Table S2.docx]

| **Table S2. Multivariable Regression Models to Predict Changes in Carotid Bulb IMT in HIV-infected Subjects** | | | |
| --- | --- | --- | --- |
| **A.** | | | |
| **Variable** | **β** | **Β (SE)** | **P** |
| Study arm* | -0.13267 | 0.05642 | 0.03 |
| CD4 count | -0.00003 | 0.00008 | 0.73 |
| ARV duration | 0.00036 | 0.00072 | 0.62 |
| PI duration | 0.00038 | 0.00077 | 0.63 |
| Sex | 0.00606 | 0.06606 | 0.93 |
| Race | 0.03328 | 0.10437 | 0.75 |
| Smoking* | -0.25887 | 0.07391 | 0.04 |
|  | R^2^ = 0.32618 | | |
| **B.** | | | |
| **Variable** | **β** | **Β (SE)** | **P** |
| Change in 25(OH)D | 0.00288 | 0.00157 | 0.08 |
| CD4 count | -0.00003 | 0.00008 | 0.70 |
| ARV duration | 0.00052 | 0.00073 | 0.48 |
| PI duration | 0.00040 | 0.00080 | 0.62 |
| Sex | 0.03486 | 0.06557 | 0.60 |
| Race | -0.02692 | 0.10497 | 0.80 |
| Smoking | -0.07995 | 0.07355 | 0.29 |
|  | R^2^ = 0.28493 | | |
| *Standard-dose arm (vs. supplementation-dose arm) and current smoking (vs. no current smoking) associated with greater decreases in carotid IMT  N.B. Supplementation dose = 60,000 IU/month (moderate dose) or 120,000 IU/month (high dose); Standard dose = 18,000 IU/month (control dose)  ARV, antiretroviral therapy; PI, protease inhibitor; 25(OH)D, 25-hydroxyvitamin D | | | |
