# Supplementary figures and images for: Effects of vitamin D supplementation on carotid intima-media thickness in HIV-infected youth
Source: Virulence. 2017 Oct 5;9(1):294–305. doi: 10.1080/21505594.2017.1365217 (PMC5955463; doi:10.1080/21505594.2017.1365217)

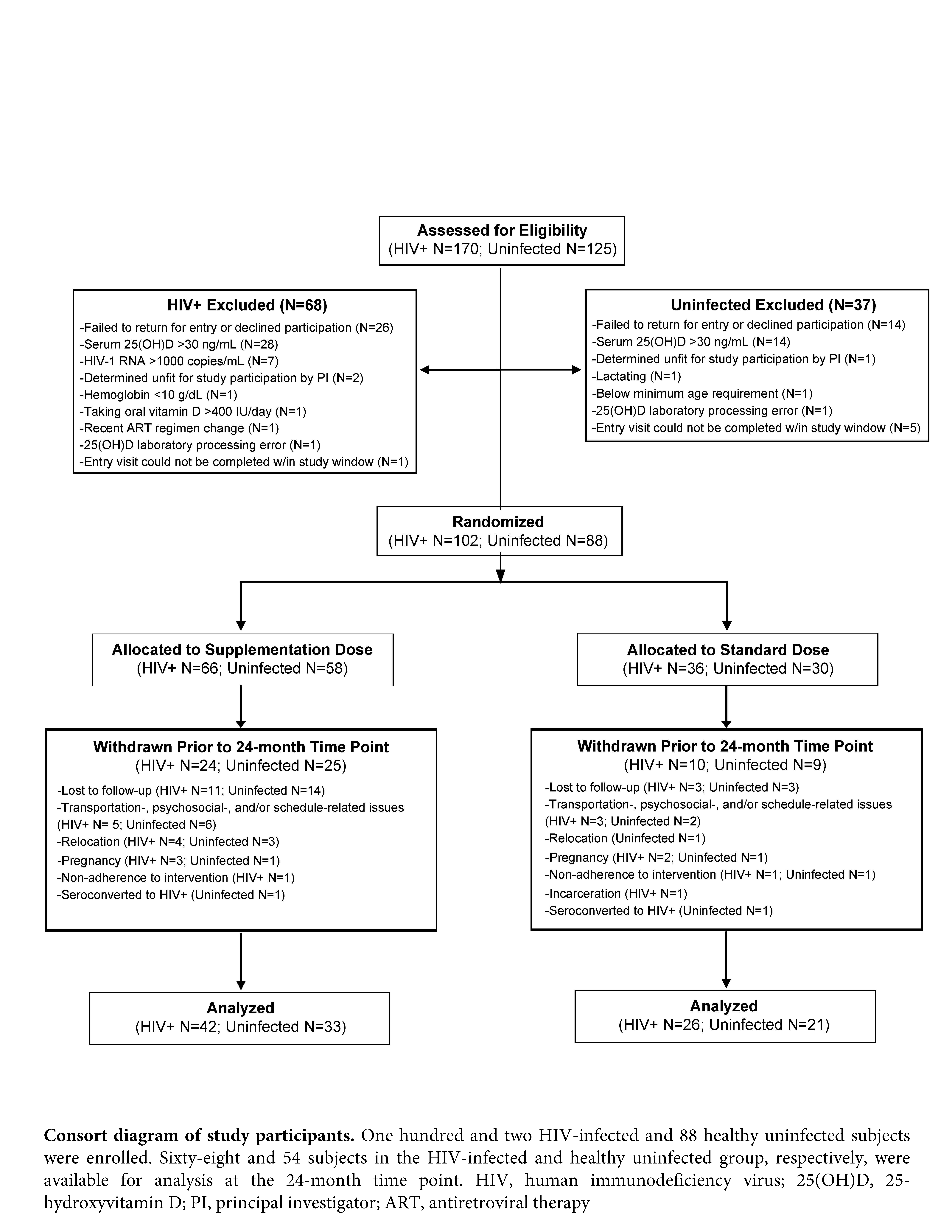

Supplement: KVIR_S_1365217.zip [file kvir-09-01-1365217-s001.zip › KVIR_S_1365217.tif]
